# Bacterial community characterization of water and intestine of the shrimp *Litopenaeus stylirostris* in a biofloc system

**DOI:** 10.1186/s12866-016-0770-z

**Published:** 2016-07-19

**Authors:** Emilie Cardona, Yannick Gueguen, Kevin Magré, Bénédicte Lorgeoux, David Piquemal, Fabien Pierrat, Florian Noguier, Denis Saulnier

**Affiliations:** Ifremer, UMR 241 EIO, UPF-ILM-IRD, Labex Corail, B.P. 7004, 98719 Taravao, Tahiti French Polynesia; Ifremer, Unité de recherche Lagons, Ecosystèmes et Aquaculture Durable en Nouvelle Calédonie, Nouméa, New Caledonia; ACOBIOM, 1682 rue de la Valsière, Cap Delta - CS77394, 34184 Montpellier Cedex 4, France; Ifremer, UMR 5244 IHPE, UPVD, CNRS, Université de Montpellier, F-34095 Montpellier, France

**Keywords:** Shrimp, Biofloc, Clear sea water, Bacterial communities, Culture environment, Microbiota

## Abstract

**Background:**

Biofloc technology (BFT), a rearing method with little or no water exchange, is gaining popularity in aquaculture. In the water column, such systems develop conglomerates of microbes, algae and protozoa, together with detritus and dead organic particles. The intensive microbial community presents in these systems can be used as a pond water quality treatment system, and the microbial protein can serve as a feed additive. The current problem with BFT is the difficulty of controlling its bacterial community composition for both optimal water quality and optimal shrimp health. The main objective of the present study was to investigate microbial diversity of samples obtained from different culture environments (Biofloc technology and clear seawater) as well as from the intestines of shrimp reared in both environments through high-throughput sequencing technology.

**Results:**

Analyses of the bacterial community identified in water from BFT and “clear seawater” (CW) systems (control) containing the shrimp *Litopenaeus stylirostris* revealed large differences in the frequency distribution of operational taxonomic units (OTUs). Four out of the five most dominant bacterial communities were different in both culture methods. Bacteria found in great abundance in BFT have two principal characteristics: the need for an organic substrate or nitrogen sources to grow and the capacity to attach to surfaces and co-aggregate. A correlation was found between bacteria groups and physicochemical and biological parameters measured in rearing tanks. Moreover, rearing-water bacterial communities influenced the microbiota of shrimp. Indeed, the biofloc environment modified the shrimp intestine microbiota, as the low level (27 %) of similarity between intestinal bacterial communities from the two treatments.

**Conclusion:**

This study provides the first information describing the complex biofloc microbial community, which can help to understand the environment-microbiota-host relationship in this rearing system.

## Background

In shrimp aquaculture, much interest has been shown for biofloc technology (BFT). BFT is a rearing system with little or no water exchange. In such a system, a conglomerate of microbes, algae and protozoa develops in the water column, along with detritus and dead organic particles [1]. The resulting intensive microbial community can serve as a pond water quality treatment system, whilst the microbial protein produced can be used as feed. The current problem with BFT is the difficulty of controlling the biofloc bacterial community composition for both optimal water quality and optimal shrimp health. Today, the research priority for BFT is to measure and describe the complex microbial biofloc community and to develop methods that will establish diverse and stable microbial communities. Few authors have quantified total heterotrophic bacteria and *Vibrio* sp. [2, 3]. Zhao et al. [4] reported that adding *Bacillus* into BFT water resulted in a decrease in *Vibrio* abundance. However, to the best of our knowledge, no author has yet characterized the totality of the bacterial communities in a BFT system.

It is assumed by many authors that, by consuming natural productivity, shrimps ingest the bacteria present on the particles [1, 5]. It is also known that the composition of aquatic bacterial communities in ponds has a strong influence on the internal bacterial microbiota of farmed marine animals; this can play a role in nutrition, immunity, and disease resistance of animals [6]. However, no study has examined the potential relationship between BFT bacteria and those of cultured animals.

Today, biofloc technology is used throughout the world. Two great advantages of BFT are that the intensive microbial community present can serve as a pond water quality treatment system whilst the microbial protein can be used as a nutrient. However, very little information is available on the bacterial communities present in the rearing water or on the interaction of this microbial environment with bacteria of the shrimp microbiota. An important step for the improvement and optimization of biofloc technology is the characterization and control of its microbial community. In this study, we characterized microbial communities of water samples obtained from different shrimp culture environments (Biofloc technology and clear seawater) as well as from the intestines of shrimp reared in both environments using MiSeq Illumina sequencing technique, and evaluated the impact of the culture environment on shrimp microbiota.

## Results and discussion

### Bacterial diversity and composition in BFT and CW rearing water

A total of 4,141,873 sequences were obtained for rearing-water with an average of 108,997 reads *per* sample (*n* = 38). Sequences were clustered into operational taxonomic units (OTUs) at the 0.97 similarity level. The Greengenes 13_8 reference OTU collection bank was used as reference. OTU richness of water varied from 435.02 ± 55.81 *per* sample in the CW treatment to 277.59 ± 23.02 in the BFT treatment.

To compare the bacterial diversity between treatments, Shannon and Simpson indices were calculated from the OTUs. The Shannon index varied from 2.86 ± 0.32 in the CW treatment to 2.66 ± 0.39 in the BFT treatment. The Simpson index varied from 0.15 ± 0.05 in the CW treatment to 0.18 ± 0.09 in the BFT treatment. The similarity in these indices suggested a similar range of diversity between the treatments. It is important to note that the Simpson index, which was close to 0, revealed a high species diversity.

Although the bacterial diversity was similar, the frequency distribution of bacterial phyla differed between the treatments (Table 1). *Proteobacteria* was the most abundant phylum in water from both treatments. In this study, its relative abundance ranged between 50.4 % for CW and 60.0 % for BFT. This phylum is widely dispersed in the marine environment and plays an important role in the process of nutrient cycling and the mineralization of organic compounds [7, 8]. *Bacteroidetes* was the second most abundant phylum, with a relative abundance ranging from 30.0 % for CW and 21.9 % for BFT. This phylum is a dominant member of marine heterotrophic bacterioplankton and is frequently found colonizing macroscopic organic matter particles [9]. Finally, *Cyanobacteria* was the third most abundant phylum, with a relative abundance ranging from 13.0 % for CW to 8.5 % for BFT. Altogether, these three phyla represented more than 90 % of the total bacteria.

**Table 1:** Relative abundance of the most frequently identified bacterial phyla (> 0.1 % of total sequence) in water from different rearing conditions: clear water (CW) and biofloc (BFT)

Bacteria phyla	CW	BFT
*Proteobacteria*	50.40	60.07
*Bacteroidetes*	30.04	21.86
*Cyanobacteria*	12.96	8.48
*Euryarchaeota*	2.77	0.00
*Actinobacteria*	2.20	2.13
*Planctomycetes*	0.36	1.58
*Verrumibrobia*	0.33	1.37
*Chloroflexi*	0.31	0.31
*Crenarchaeota*	0.17	0.00

In BFT water, *Leucothrix* (20.1 %), *Rhodobacteraceae* (16.4 %), *Stramenopiles* (8.0 %), *Oceanospirillaceae* (5.5 %) and *Saprospiraceae* (4.7 %) were the most dominant operational taxonomic units. In the CW conditions, *Cryomorphaceae* (24.6 %), *Pelagibacteraceae* (10.1 %), *Stramenopiles* (8.4 %), *Glaciecola* (5.6 %) and *Colwelliaceae* (4.9 %) were the most represented taxa.

**Fig. 1:**
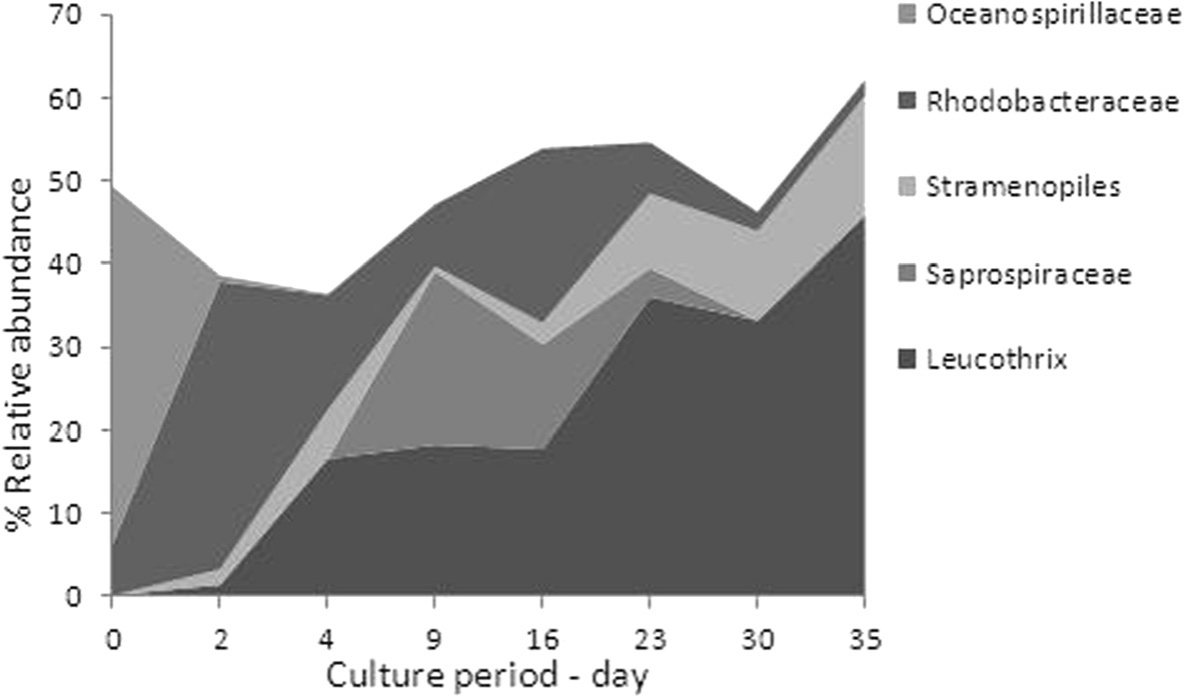
Changes in relative abundance of the five main taxa over the course of rearing in BFT

**Fig. 2:**
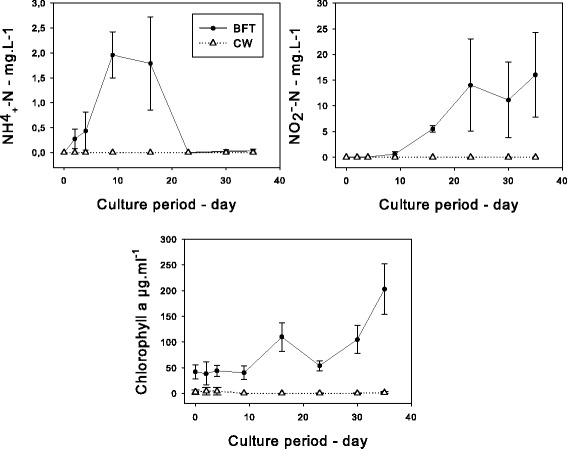
Evolution of NO_2_^−^-N, NH_4_^+^-N and Chlorophyll a concentrations over the course of rearing

A high level of variability in the most frequent bacterial communities was evidenced over the course of BFT rearing (Fig. 1) that could be due to changes in biotic and abiotic environmental factors [10]. In the BFT system, several physicochemical and biological parameters such as chlorophyll A (Chl a), nitrite nitrogen (NO_2_^−^-N) and total ammonia nitrogen (NH_4_^+^-N) changed over time, whereas these parameters were stable and close to zero in the CW system (Fig. 2). In the BFT system, a classic sequence of nitrogen species was observed as described by [1]: when a zero exchange water tank is stocked and fed, processes start that lead to an ammonium build up in the water. When ammonium concentration rises, the ammonium oxidizing bacteria population starts to develop and oxidize the evolved ammonium, leading to the lowering of NH4^+^-N concentration. At this point, nitrite oxidizing bacteria start to develop. During this transition period, nitrite concentration in the water rises. Then, when there are enough bacteria, they control both the ammonium and nitrite concentrations. pH values were relatively stable over time (between 7.7 and 7.9). A correlation was detected between the evolution of the major bacterial groups found in BFT water and three environmental parameters of rearing (NO_2_^−^-N, NH_4_^+^- N and Chl a) using Spearman’s test. The frequency distribution of *Leucotrix* increased with the time, peaking at the last rearing day at 40.2 % of the total bacteria. This genus is considered to be comprised of bacteria capable of filamentous growth [11]. Its evolution was positively correlated with nitrite concentration (*p* value < 0.0001). Nitrite could be used as a growth source by this genus. Moreover, this genus needs to be attached to survive. In BFT, the high concentration of biofloc particles in the water column can serve as mounting surfaces (222 ± 85 mg.L^−1^). Moreover, *Leucotrix mucor*, the most studied species in this genus, was found to obtain its nutrients for growth from algae rather than from seawater [11]. It is therefore logical that its evolution is significantly correlated with Chl a concentration (*p* = 0.004). However, *Leucotrix mucor* is known to cause fouling disease in shrimps, although it is not considered to be a true pathogen [12]. The family *Oceanospirillaceae* representing 43.1 % before the beginning of the experiment, decreased significantly after 2 days of rearing (1.2 %). The genus that displayed the greatest change after the beginning of the experiment was *Marinomonas*. The abrupt change could be attributed to the large increase in shrimp biomass between the start of the experiment and the point at which the biofloc was well developed. Quantities of *Bacteroidetes Saprospiraceae*, bacteria found only in BFT water, were positively correlated with NH_4_^+^-N concentration (*p* > 0.0001). This taxon can easily use the organic substrate [13]. *Rhodobacteraceae*, found at a high frequency in the BFT water, is frequently detected in aquaculture biofilm systems [14, 15]. It is considered an excellent biofilm-forming organism and is among the first and dominant colonizers of the surface in all marine environments [16, 17]. The ability to effectively colonize surfaces gives *Rhodobacteraceae* a competitive advantage in the BFT, where total suspended solid concentration is important. In addition, the *Rhodobacteraceae* family is known to exhibit a diverse range of metabolic activity. The *Rhodobacter* group can possibly establish an antagonistic beneficial bacterial community in the rearing environment of turbot larva and thereby limit the survival of pathogenic bacteria [15]. In our study, *Rhodobacteraceae* may therefore have played an important role in maintaining the health of the culture system. Indeed, the *Vibrionales* order represented 6 % of total sequence in CW conditions (1.2 % *Pseudoalteromonadaceae* and 4.7 % *Vibrionaceae*), whereas it represented only 1.5 % in BFT conditions (1.4 % *Pseudoalteromonadaceae* and 0.1 % *Vibrionaceae*). *Vibrio*, belonging to the *Vibrionaceae* family, has been characterized as pathogens for some aquatic organisms including crustacean larvae and juveniles [18–25]. Because *Vibrio* is less abundant in BFT (0.01 % in BFT water *vs* 0.73 % in CW water), shrimps could be less exposed to vibrioses. In the CW treatment, 80.2 % of the total *Vibrionaceae* sequence was represented by *Photobacterium damselae*. This species is known to be a marine fish pathogen that can be transmitted to fish through water [26]. It seems that BFT limited the proliferation of this species and could thus minimize the risk of infection. In this study, the *Rhodobacteraceae* family could have limited the survival of pathogenic bacteria in the BFT compared with the CW environment. Another suggestion to explain the lower *Vibrio* abundance in BFT is that bioflocs displayed biocontrol activity against *Vibrio harveyi,* the virulence of which can be regulated by the process of cell-to-cell communication called quorum sensing [27, 28].

In this work, the CW environment harbored communities of bacterial genera that were distinct from those found in the BFT environment. Indeed, results from similarity percentages revealed that profiles of water samples from each treatment were distinct, with 89 % dissimilarity. Similarity percentage analysis (SIMPER) showed that the genera *Photobacterium*, *Leucothrix* and *Vibrio* were the three principal taxa at the root of the dissimilarity (16.5, 12.1 and 4.1 %, respectively) between water samples from the two treatments. Among the most represented taxa found in CW environment, *Pelagibacteraceae* is already known to constitute a family of extreme oligotrophs that are widely distributed across the Earth’s oceans [29]. Moreover, *Alteromonadacea*, *Cryomorphaceae* and *Pelagibacteraceae* families were considered the most abundant bacterial groups in the eastern Mediterranean Sea, which is an ultraoligotrophic marine habitat [30]. Their significant presence in the CW water can be explained by the fact that water was pumped directly from the lagoon of Tahiti (French Polynesia), which is also oligotrophic seawater. In the CW tank, water renewal was very high (300 % *per* day); it is therefore logical that the bacterial communities in the tank reflect those of the lagoon.

Based on these observations, we can explain the difference in bacterial communities between the BFT and CW environments by the high levels of organic matter and nutrient concentrations present in biofloc. The biofloc treatment was characterized by bacteria belonging to taxa such as *Gammaproteobacteria Thiotrichaceae Leucothrix*, *Alphaproteobacteria Rhodobacteraceae* and *Bacteroidetes Saprospiraceae*. These bacteria have the particularity of using organic matter and nitrogen compounds for growth and require a support to grow on. These two conditions are found in the BFT system, which is particularly rich in organic matter and suspended particles in the water column. This ability to attach to the surfaces and to use organic matter may be a significant physiological characteristic of bacteria in biofloc. In contrast, the bacterial groups found in the CW system, mainly *Alteromonadaceae*, *Cryomorphaceae* and *Pelagibacteraceae* families, are characteristics of oligotrophic waters.

### Effect of rearing environment on shrimp microbiota

In aquatic ecosystems, the close relationships between microorganisms and other biota and the constant flow of water through the digestive tract of fish and invertebrates will also affect their indigenous microflora [31]. In this study, we focused on the bacterial distribution and composition in shrimp intestine after 35 days of rearing according to different conditions, CW or BFT.

A total of 963,352 sequences were obtained for intestine samples, with an average of 118,843 reads *per* CW sample (*n* = 3) and 151,702 reads *per* BFT sample (*n* = 4). The OTU richness of intestine samples varied from 239.00 ± 31.11 *per* sample in the CW treatment to 196.25 ± 9.00 in the BFT treatment. The relative abundance of major order bacteria in the intestine samples from both types of growing conditions is presented in Table 2. It should be remembered that the shrimps had not eaten any commercial feed for 24 h before sampling. BFT and CW libraries from intestine samples were mostly dominated by one order affiliated to *Gammaproteobacteria Vibrionales*. This order is better represented in intestines from CW-reared shrimps (69.2 % of total sequences) than those from BFT-reared shrimps (58.6 % of total sequences). This group of bacteria has previously been reported at high relative abundance in the intestines of other species of shrimps such as *Litopenaeus vannamei, Peneaus monodon* and *Fenneropenaeus chinensis* [32–35]. *Alphaproteobacteria Rhodobacterales* was the second most abundant order, with a relative abundance ranging from 13.0 % for CW and 2.0 % for BFT. *Chloroplast Stramenopiles* only represented a small fraction of the sequences, but showed a difference between the treatments: 10.5 % in CW shrimp intestines, but less than 2 % in BFT shrimp intestines. *Flavobacteriia Flavobacteriales* and *Bacteroidia Bacteroidales* orders belonging to the *Bacteroidetes*, and *Mollicutes* orders belonging to the phylum *Tenericutes* represented 5.5, 6.7 and 6.0 %, respectively, in BFT shrimp intestines, while these orders represented less than 2 % in CW shrimp intestines.

**Table 2:** Relative abundance of the most frequently identified bacterial orders (> 1 % of total sequences) within shrimp intestines from two different types of rearing conditions: clear water (CW) or Biofloc (BFT)

Taxa	CW	BFT
*Gammaproteobacteria Vibrionales*	69.18	58.60
*Alphaproteobacteria Rhodobacterales*	12.99	2.02
*Chloroplast Stramenopiles*	10.53	0.14
*Bacteroidia Bacteroidales*	0.01	6.74
*Flavobacteriia Flavobacteriales*	1.87	5.53
*Gammaproteobacteria Alteromonadales*	1.57	1.11
*Mollicutes*	1.29	5.95

The *Pseudoalteromonadaceae* family represented 8.2 and 0.8 % in the shrimp intestines from the CW and BFT treatments, respectively. The *Vibrionaceae* family represented 57.6 and 54.5 % in shrimp intestine from the CW and BFT treatments, respectively. *Photobacterium* represented 82 to 89 % of *Vibrionaceae* in the total sequence from all the shrimp intestines, while *Vibrio* accounted for the remainder.

The Shannon index varied from 1.91 ± 0.67 in the CW treatment to 2.01 ± 0.68 in the BFT treatment, and the Simpson index varied from 0.32 ± 0.19 in the CW treatment to 0.26 ± 0.15 in the BFT treatment. These comparable indices suggested a similar range of diversity in the two treatments. However, compared with indices measured in water, bacterial diversity estimated by the Shannon index, in which a higher value means greater bacterial diversity, revealed a significantly lower diversity in the intestine compared to water (*p* < 0.0001). Similarly, the Simpson index, for which a lower value means a greater bacterial diversity, confirmed this result (*p* < 0.0001). Johnson et al. [36] showed that water had substantially more diverse microflora than the intestine of shrimps. They expected this because the environment of the shrimp gut is different from water in an aquaculture tank and not all microbes in the water would be ingested and survive in the gut. Moreover, shrimp gut is less aerobic than tank water and has immunological factors which may differentially select against some bacteria.

**Fig. 3:**
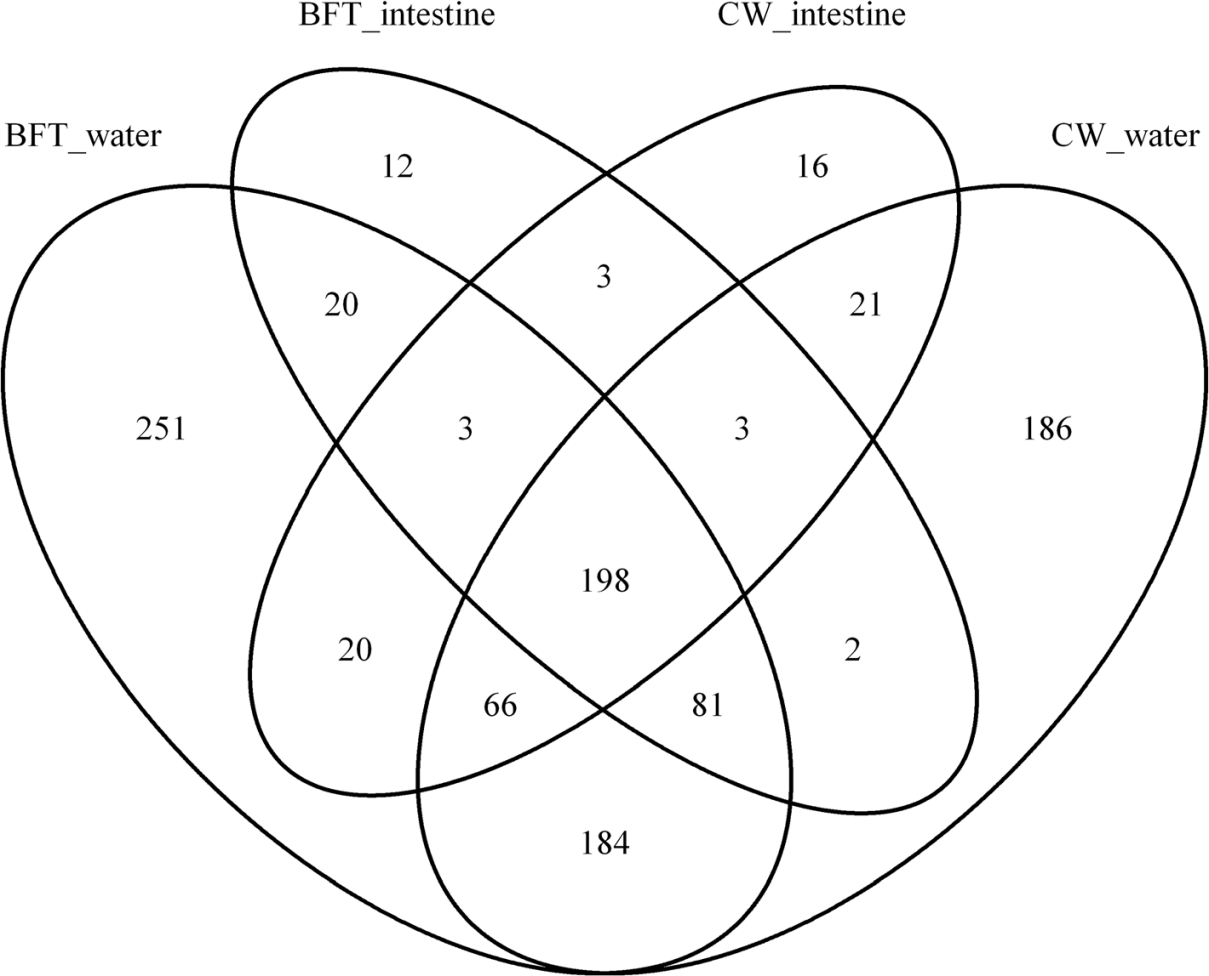
Venn diagram showing the unique and shared OTUs (3 % of distance level) in the different libraries: BFT and CW intestines, BFT and CW water

Bacterial OTUs in rearing water and shrimp intestine were investigated with regard to treatment and are shown quantitatively in the Venn diagram in Fig. 3. The analyses revealed that 198 OTUs were shared between all sample types, which corresponds to 10.7 % of the total OTUs. The diagram clearly emphasizes that water samples have a large pool of OTUs that are not found in the intestines [251 (24.5 %) and 186 (28.2 %) in water from BFT and CW, respectively, with 184 OTUs (33.6 %) in common], which contrasts with the low number of OTUs found exclusively in intestine [12 (1.1 %) and 16 (2.3 %) from BFT and CW, respectively, 3 OTUs (1.5 %) in common]. This result confirmed that the flora found in water is substantially more diverse than that of shrimp guts.

**Fig. 4:**
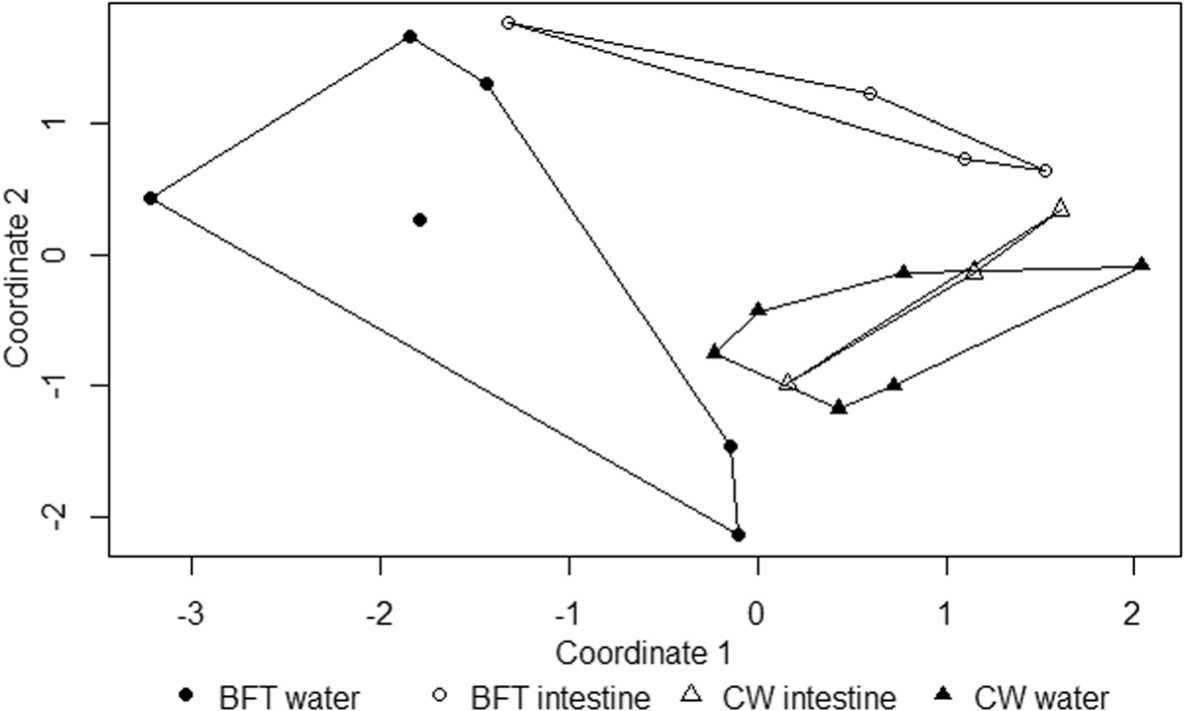
Non-metric multidimensional scaling (MDS) plot using the Bray-Curtis coefficient of sequencing results associated with intestines and water samples from CW or BFT treatments after 35 days of rearing. Each point represents a sequencing profile from one sample

Significant differences in the bacterial communities of intestines and water from CW and BFT treatments were visualized by the multidimensional scale (MDS) plot, and confirmed using analysis of similarity (ANOSIM) (*p* = 0.002) (Fig. 4). A stress value below 0.2 (0.14 in our case) indicates that an MDS ordination plot is a good spatial representation of the difference in the data. ANOSIM analysis takes into account the difference of bacterial taxa (OTUs) and their relative abundance, in contrast to a Venn diagram, which only compares the number of bacterial OTUs between the different growing conditions. In this figure, bacterial communities in CW intestines reflected the outside CW environment after one month of rearing. Moreover, shrimp intestines from BFT had bacterial communities distinct from those in CW.

Previous investigations have proposed that the intestinal microbiota of shrimp originate from their cultural environment [33, 37, 38]. In the present study, profiles of intestinal bacterial communities from each treatment were distinct (only 27 % similarity). Moreover, results of the MDS analysis showed that the composition of bacterial communities in shrimp intestines from the CW treatment was relatively similar to those of the rearing environment. Similarity percentage analysis (SIMPER) revealed 35 % similarity between CW water and intestine samples. However, this was not the case for the BFT treatment, as similarity percentage analysis revealed only 13 % similarity between water and intestine samples. According to the SIMPER analysis, *Cryomorphaceae* sp., *Photobacterium damselae* and *Pelagibacteraceae* were the three principal taxa at the root of dissimilarity (13.6, 13.3 and 6.5 %, respectively) between the intestine samples from both treatments. Interestingly, these taxa were found at a greater frequency in the CW intestine and its corresponding water compared to BFT intestine and its corresponding water, confirming the influence of the bacterial composition of the water environment on that of the intestine. *Leucothrix* and *Rhodobacteraceae* contributed 2.9 and 1.8 %, respectively, to the dissimilarity. Conversely, these group abundances were greater in BFT intestines and water than in CW intestines and water. These results also suggest that the bacterial composition of BFT water influenced that of the intestine. Nonetheless, BFT intestine bacteria had specificities different from those of CW intestine. Indeed, the MDS plot revealed differences in bacterial specificity between BFT intestine and BFT water, whereas for the CW treatment, intestine bacteria communities reflected those of rearing water more closely.

## Conclusion

The present study explored the diversity of bacterial communities by applying 16S RNA next-generation sequencing to different culture environments (BFT and CW) and intestines of shrimp reared in both environments. The results demonstrated, first, that the water harbored a remarkable diversity of bacterial communities and that four out of the five most important bacterial communities were different between culture methods. Second, this study showed that the culture environment interacted with the intestinal microbial communities. This swork provides descriptive information about a bacterial community associated with biofloc rearing, and its influence on shrimp intestinal microbiota, which can further be applied to research on immunity, disease resistance and nutrition of shrimp in aquaculture.

## Methods

### Experimental design

Before the experiment, the biofloc culture was established in one 25-m^2^ tank with a low shrimp biomass of 300 g.m^−2^ for three months. Daily water renewal was only 3 % of the tank volume, done mainly to remove deposits of excess organic matter (ecdysis, dead animals, unconsumed pellets, etc.). Shrimps (*Litopenaeus stylirostris*) were fed twice a day with commercial shrimp feed (SICA^(c)^ grower 40 % protein). The tank was continuously aerated with bubbled air delivered through a stone diffuser and was covered with a shade net to limit sunlight (70 % inhibition of light). After this period, the biofloc was transferred into 12 outdoor polyester tanks (capacity, 250 l). Juveniles of shrimps (4.45 ± 1.27 g) were randomly assigned for 35 days to two types of growing conditions, both of which had an initial mean shrimp biomass of 900 g.m^−2^: Clear seaWater system (CW) and BioFloc Technology (BFT). The experiment was conducted in 18 outdoor 250-L polyester tanks in the *Centre Technique Aquacole de Vairao,* French Polynesia [Aquaculture Technical Center of Tahiti], with 6 replicates for CW and 12 replicates for BFT. Each tank was continuously aerated with blown air delivered through a stone diffuser, and was covered with a shade net to limit sunlight (70 % inhibition of light). The water renewal rate was 300 % per day in the CW treatment and 3 % in BFT; water was directly pumped from a lagoon in Tahiti (French Polynesia). To balance the Carbon:Nitrogen (C:N) ratio and encourage the development of heterotrophic bacteria in BFT, sugar cane molasses was added daily to obtain a C:N of 20:1. The calculation of C:N ratio takes into account the nitrogen content in feed, the quantity of food distributed and the rate of nitrogen excretion by the shrimp as mentioned in the manual on the biofloc culture method [1]. Shrimp were fed twice a day (8 am and 3 pm) with commercial shrimp feed (SICA^(c)^ grower 40 % protein).

### Physicochemical and biological measurement

Temperature and dissolved oxygen were recorded twice a day (08:00 am and 03:00 pm) with an OxyGard Handy Gamma probe. The pH was recorded once a day (08:00 am) with a pH meter (Hach Lange HQ 40D). Furthermore, total ammonia nitrogen (NH_4_^+^-N) and nitrite nitrogen (NO_2_^−^-N) were analyzed twice a week by a fluorescence method, according to [39], and a spectrophotometric method, according to [40]. Chlorophyll a (Chl a) was determined using a spectrophotometer (Trilogy Turner Design) at 664 and 750 nm wavelengths, following the method of [41].

### Water and intestine sample collection

Once a week, 60 mL of BFT rearing water were sampled *per* tank and filtered through a 0.22-μm Sterivex Millipore filter. CW rearing water was sampled in the same manner but only twice during rearing period; we considered that with the higher water renewal (300 % *per* day), bacterial communities did not change during rearing and assumed that the composition and diversity of original influent was very similar to that of the CW rearing system. The filter was put into 2-mL microcentrifuge tubes with 1 mL of lysis solution (0.1 M EDTA- pH 8; 1 % SDS and 200 μg.ml^−1^ proteinase K) and incubated at 55 °C for 24 h.

At the end of the experiment, shrimps were not fed for 24 h before sampling. Three shrimps were then randomly sampled from each tank; their intestines were aseptically dissected and put directly into 1.5-mL microcentrifuge tubes with 0.5 mL of lysis solution. These samples were then incubated for 24 h at 55 °C.

### DNA extraction

CTAB extraction was performed on the lysis extract to remove both polysaccharides and residual proteins [42]. Briefly, 165 μl of pre-warmed 10 % CTAB and 165 μl of 5 M NaCl were added to each 0.5 ml volume of lysis extract. After vortexing, samples were incubated at 55 °C for 10 min. Nucleic acids were extracted by a classic method using Phenol: Chloroform: Isoamyl Alcohol (25:24:1), then precipitated with one volume of isopropanol. After centrifugation at 10 000 *g* and 4 °C for 5 min, pelleted DNA was washed with 70 % ethanol to remove salts, dried under vacuum and resuspended in 50 μL ultrapure water. The concentration of the extracted DNAs, stored at −20 °C, were tested using the NanoDrop® (ND-1000 Spectrophotometer) and their purity assessed by the analysis of the ratio of absorbance at 260 nm and 280 nm (OD 260/OD 280).

### PCR amplification of 16SrNA genes and next-generation sequencing

A barcoded sequencing approach was used to study the bacterial composition of each water and intestine samples. PCR amplification was performed prior to sequencing. All of the PCR components were UV-irradiated for 30 min under a UV lamp prior to the addition of Taq polymerase and DNA. The primer pair “V4 16S rRNA F 515” (5′ GTG CCA GCM GCC GCG GTA A3′) and “V4 16SrRNA R 806” (5′GGA CTA CHV GGG TWT CTA AT 3′), targeting the V4 variable region of the 16S rRNA gene [43], was used to amplify the DNA samples by PCR (PTC-100, MJ Research, Inc.). The Go Taq Hot Start Polymerase Promega kit was used following the manufacturer’s recommendations. Briefly, PCR was carried in 1X PCR amplification buffer (10X buffer contains 200 mM TRIS HCl; pH 8.4), 500 mM KCl) and 2 mM MgCl_2_ with 0.5 μM of each primer, 0.2 mM of each deoxyribonucleotide (dNTP), 1 μL DNA suspension and 1.25 U Taq DNA polymerase (Go Taq Promega™ 5 U/ml)). After initial denaturation at 95 °C for 5 min, 30 cycles of amplification were carried out starting at 95 °C for 1 min, followed by 30 s at 58 °C, and 1 min at 72 °C, with a final extension at 72 °C for 5 min. The quality of PCR products was verified by migration on a 1 % agarose electrophoresis gel. The samples were then sent to be analyzed at the Acobiom laboratory (Montpellier, France). Libraries were generated by the MiSeq Illumina genome sequencer (300 cycles).

### Data analysis

Data were analyzed using the Qiime 1.8.0 software package (Quantitative Insights Into Microbial Ecology). First, data were filtered to remove forward primers, reverse primers and barcode sequences. Operational Taxonomic Units (OTUs) were selected (97 % similarity) using the pick_closed_reference_otus.py script with the uclust method and Greengenes 13_8 reference. Shannon and Simpson indices were then determined. An OTU table of the samples was generated for downstream analysis with R Studio v0.98.1091. A Venn diagram was created to determine specific and common bacterial species of the different samples. A non-metric multidimensional (MDS) plot using the Bray-Curtis similarity index was used to visualize the differences between samples. Analysis of similarities (ANOSIM) was performed to test whether there were significant differences between different groups. Moreover, similarity percentage analysis (SIMPER) was used to define the taxon at the root of dissimilarity between sample types. Frequency distribution matrices of the sample sequencing results were used for these three types of analysis. Correlations between the evolution of the major bacterial genera found in BFT water and environmental parameters of rearing were tested using the critical value table for Spearman’s rank correlation coefficient rho at the 5 % alpha level.

## Abbreviations

ANOSIM, analysis of similarity; BFT, biofloc technology; C:N, Carbon:Nitrogen; Chl a, Chlorophyll a; CW, clear sea water; MDS, multidimensional scaling; NH_4_^+^-N, total ammonia nitrogen; NO_2_^−^-N, nitrite nitrogen; OTU, operational taxonomic unit; PCR, polymerase chain reaction; SIMPER, similarity percentage
